# Y_MAP_: a pipeline for visualization of copy number variation and loss of heterozygosity in eukaryotic pathogens

**DOI:** 10.1186/s13073-014-0100-8

**Published:** 2014-11-20

**Authors:** Darren A Abbey, Jason Funt, Mor N Lurie-Weinberger, Dawn A Thompson, Aviv Regev, Chad L Myers, Judith Berman

**Affiliations:** Department of Genetics, Cell Biology and Development, University of Minnesota, 6-160 Jackson Hall, Minneapolis, MN 55415 USA; Broad Institute of MIT and Harvard University, 415 Main Street, Cambridge, MA 02142 USA; Department of Molecular Microbiology and Biotechnology, Tel Aviv University, 418 Britannia Building, Ramat Aviv, 69978 Israel; Department of Computer Science and Engineering, University of Minnesota, 200 Union St SE, Minneapolis, MN 55455 USA

## Abstract

The design of effective antimicrobial therapies for serious eukaryotic pathogens requires a clear understanding of their highly variable genomes. To facilitate analysis of copy number variations, single nucleotide polymorphisms and loss of heterozygosity events in these pathogens, we developed a pipeline for analyzing diverse genome-scale datasets from microarray, deep sequencing, and restriction site associated DNA sequence experiments for clinical and laboratory strains of *Candida albicans*, the most prevalent human fungal pathogen. The Y_MAP_ pipeline (http://lovelace.cs.umn.edu/Ymap/) automatically illustrates genome-wide information in a single intuitive figure and is readily modified for the analysis of other pathogens with small genomes.

**Electronic supplementary material:**

The online version of this article (doi:10.1186/s13073-014-0100-8) contains supplementary material, which is available to authorized users.

## Background

The collection of large, near-comprehensive genomic datasets of human pathogens such as *Candida albicans* has become common due to the availability of next-generation sequencing technologies. A major challenge is to represent these large, complex datasets that probe a heterozygous diploid genome in a manner that is biologically relevant and easy to interpret. In *C. albicans*, genome changes of small scale (single nucleotide polymorphisms (SNPs), short insertions, and short deletions) and large scale (duplications, deletions, loss of heterozygosity) can have important consequences in the development of new clinical phenotypes, most notably, drug resistance [1,2].

The *C. albicans* genome has eight linear chromosomes that are highly heterozygous (approximately 70K SNPs between homologs), compact (0.9 to 3.2 Mbp) and are not detectable via microscopy-based karyotyping methods. Contour-clamped homogenous electric field (CHEF) electrophoresis provides information on relative chromosome sizes but is time consuming, low throughput, and not definitive without additional Southern blot analyses of individual probes for different chromosome regions. Thus, whole genome analyses via microarrays, deep sequencing, or sequence sampling methods, such as double-digest restriction-site associated DNA sequencing (ddRADseq), have the potential to improve the speed and precision of genome analysis.

Mapping of small yeast genomes was pioneered in *Saccharomyces cerevisiae*, which has 16 very small chromosomes (0.2 to 1.5 Mbp), point centromeres spanning only approximately 100 bp and short telomere repeats that span approximately 300 to 400 bp, a single rDNA locus containing approximately 150 tandem repeats, and no other major regions of repetitive DNA [3]. *C. albicans*, like higher organisms, has regional, epigenetic centromeres that are relatively small (3 to 5 kbp compared with 0.5 to 10 Mbp in humans) [4,5], telomere repeats that span several hundred base pairs [6] and a set of telomere-adjacent genes (*TLO1* to *TLO16*) found at most chromosome ends [7,8]. In addition to the single rDNA locus that includes 25 to 175 tandem repeats, *C. albicans* chromosomes each carry one or two major repeat sequences composed of nested repeat units that span 50 to 130 kbp [9,10]. Several different categories of transposons and long terminal repeats are also scattered throughout the chromosomes. In *C. albicans*, as in human cancer cells and some normal human tissues, aneuploid chromosomes appear frequently and in some cases specific aneuploidies or genome changes are diagnostic of specific changes, such as the acquisition of drug resistance [1,11]. Thus, the ability to detect karyotype changes in the *C. albicans* genome can facilitate informed choices regarding therapeutic strategies.

Most available tools for genome analysis were designed primarily to analyze human genome sequence data and assist in disease diagnosis. Many tools identify short-range variations in next-generation sequence datasets (reviewed in [12,13]). Most tools that produce a visualization primarily represent one major aspect of a genome: rearrangements (for example, CIRCUS [14], inGAP [15], Gremlin [16]) or large CNVs (WISECONDOR [17], FAST-SeqS [18]). Few tools provide a whole genome view of the calculated genome changes in a single glance/figure. ChARM [19] detects and visualizes copy number changes in microarray datasets. CEQer [20] and ExomeCNV [21] process and visualize copy number changes in exome-only sequence data. One of the most versatile visualization tools, IGV [22,23], can display different types of genomic variants (for example, copy number variation (CNV), SNPs, loss of heterozygosity (LOH), sequence coverage, among others), but visualization is limited to one genomic phenotype at a time, and thus it is not readily applied to time series data. Further, when applied across the entire genome view, as opposed to single chromosome views, other genomic features (that is, centromeres, telomeres, repetitive sequence elements) are not displayed.

Here we present Y_MAP_, a genome analysis pipeline motivated by the need to analyze whole genome data in a manner that provides an overview of the entire genome, including major changes in CNVs and allele ratios (LOHs) that it has undergone. As such, Y_MAP_ utilizes and extends existing tools for both short- and long-range genome analyses to provide a whole-genome view of CNVs and LOHs in small genomes, using *C. albicans* as a test case. Y_MAP_ is designed to be amenable to the analysis of clinical as well as laboratory isolates and to be readily adapted for the study of genome organization in other pathogenic yeast species. For genomes with known haplotypes, Y_MAP_ utilizes a color scheme to visualize the allele specificity of segmental and whole chromosome LOHs. For new genomes such as clinical isolates, it visualizes LOH events and, with appropriate homozygosed derivatives, it facilitates the construction of haplotype maps (hapmaps) [24]. Originally designed to process microarray data that include both SNP and comparative genomic hybridization (CGH) data [25], Y_MAP_ accepts several types of whole genome datasets. Y_MAP_ processes paired- and single-end whole genome sequence, as well as paired- and single-end ddRADseq data, which samples a sparse number of genomic loci at low cost per sample [26]. Dense histograms indicate DNA copy number and color schemes provide allele status information with data plotted either vertically for an individual strain or horizontally to facilitate comparison between individuals.

The Y_MAP_ website is available for use at [27] and includes some example datasets as well as decision flow-diagrams to help determine if the pipeline will be able to process your data (Additional file 1). The source files and directory organization needed for installing the pipeline on your own server can be downloaded from [28].

## Implementation

**Figure 1:**
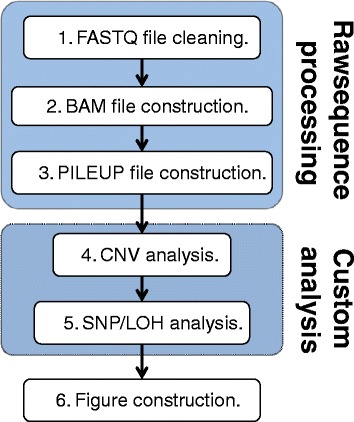
**Conceptual overview of Y**_**MAP**_ **genome analysis pipeline.** The central computation engine of the pipeline has three major components: raw sequence processing, custom analysis, and figure construction/presentation.

The genome analysis pipeline is composed of three main components: a module that performs raw sequence alignment and processing (Figure 1, steps 1 to 3), a module that performs custom CNV and SNP/LOH analyses, and a module that constructs figures summarizing all completed analyses and then displays them on the webpage. The implementation details for each of these components are described in more detail in the following sections. The accession numbers for the sequence data for strains analyzed can be found at NCBI (BioSample accessions 3144957 through 3144969).

The first component of the central computation engine takes the user-input data and attempts to correct some typical file errors before outputting corrected FASTQ file(s) for use by later steps in the pipeline. Typical sequence data are input as one or two (for paired-end reads) FASTQ format files, either raw or compressed in the ZIP or GZ format. Depending on connection reliability, uploading a 500 Mb compressed file can take from minutes to a few hours. The large size of FASTQ files leaves them prone to file transfer errors that result in corruption because the file format does not have an internal error correction/identification system. This corruption often results in the final read entry being incomplete, which can cause analysis programs to crash, and normally has to be dealt with on a case-by-case basis. The size of the uploaded file is available in the ‘Manage Datasets’ tab beside the dataset name. Users can thus manually check whether the uploaded file size is equal to the expected file size. The issue of transfer errors is partially dealt with internally by trimming the FASTQ file to remove incomplete entries. Trimming the longer of the paired-end FASTQ files to the length of the shorter file is also done to deal with single-end reads that are generated by some sequencing technologies. Both steps are done through in-house scripts (available at [28]; incomplete entry removal: sh/FASTQ_1_trimming.sh or unbalanced reads: sh/FASTQ_2_trimming.sh).

The second step in the central computation pipeline is to process the corrected FASTQ file into a final Binary sequence Alignment/Mapping (BAM) file. The single- or paired-end reads are aligned to one of the installed reference genomes using Bowtie2 with SAM output mode set to ‘very sensitive’ [29], resulting in a Sequence Alignment/Mapping (SAM) file. SAMtools [30] is used to compress this into a BAM file. PicardTools [31] is used to standardize the read-group headers in the BAM files, to resolve some formatting irregularities to the BAM file. SAMtools is then used to sort the BAM file, which is required for efficient later processing steps. FASTQC [32] is used to identify the quality coding system used in the input FASTQ files, as a prelude to defining the input parameters for processing by the Genome Analysis ToolKit (GATK) [33], which performs indel-realignment of the BAM files, removing spurious apparent SNPs around true indels in the primary alignment. Settings for all outside tools can be found in the source code on sourceforge [28] by looking at the sh/project.paired_*.sh and sh/project.single_*.sh shell scripts.

The third step in the sequence data processing component of the pipeline is to convert the BAM file into a simpler text file containing limited data for each coordinate across the genome, which simplifies later processing. The SAMtools function mpileup first processes the BAM file into a ‘pileup’ file, which contains information about all of the mapped reads at each chromosome coordinate in a simple format that facilitates subsequent processing by custom Python scripts (available at [28] in the ‘py’ directory). The Python scripts extract base call counts for each coordinate, discarding indel and read start/end information. The raw read-depth data per coordinate is saved to a text file [‘SNP_CNV.txt’] that is input into the CNV analysis section of the pipeline. Any coordinates with more than one base call have that information saved to a separate text file [‘putative_SNPs.txt’] that is input into the SNP and LOH analysis section of the pipeline. These two files can be downloaded after being made in the ‘Manage Datasets’ tab by selecting either ‘SNP_CNV data’ or ‘putative_SNP data’ beside the relevant dataset name.

Detailed flow diagrams explaining the processes each file goes through upon introduction to Y_MAP_ are available in Additional files 2, 3, 4, and 5.

### Copy number variation analysis

**Figure 2:**
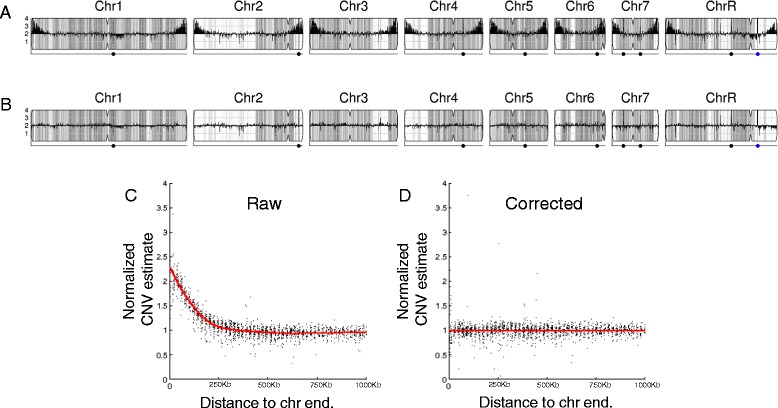
**Normalization of chromosome-end bias. (A,** **B)** Black bars up- and down-wards from the figure midline represent local copy number estimates, scaled to genome ploidy. Different levels of grey shading in the background indicate local changes in SNP density, with darker grey indicating more SNPs. Detailed interpretations are similar to those described in [25]. **(A)** Map of data with chromosome end bias present in read-depth CNV estimates for strain YQ2 dataset (from EMBL-EBI BioSamples database [34], accession SAMEA1879786). **(B)** Corrected CNV estimates for strain YQ2 mapped across all *C. albicans* chromosomes. **(C,** **D)** Raw and corrected normalized read-depth CNV estimates relative to distance from chromosome ends. Red, LOWESS fit curve.

**Figure 3:**
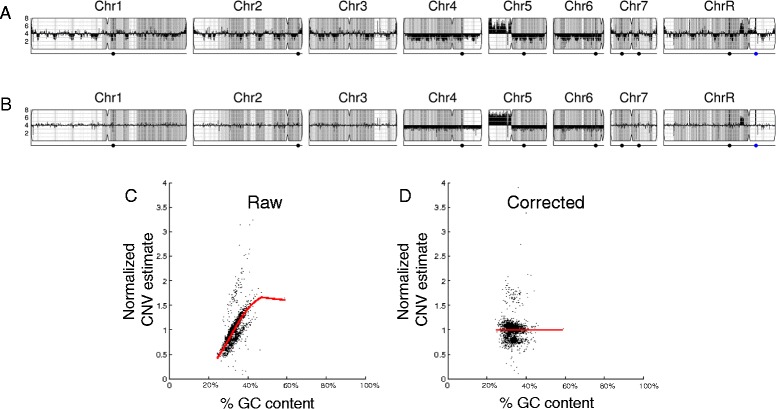
**Normalization of GC-content bias. (A)** GC-content bias present in read-depth CNV estimates using WGseq for strain FH6. **(B)** Corrected CNV estimates mapped across FH6 genome. **(C**,**D)** Raw and corrected normalized read-depth CNV estimates versus GC content. Red, LOWESS fit curve. Chromosome illustrations are as in Figure 2.

**Figure 4:**
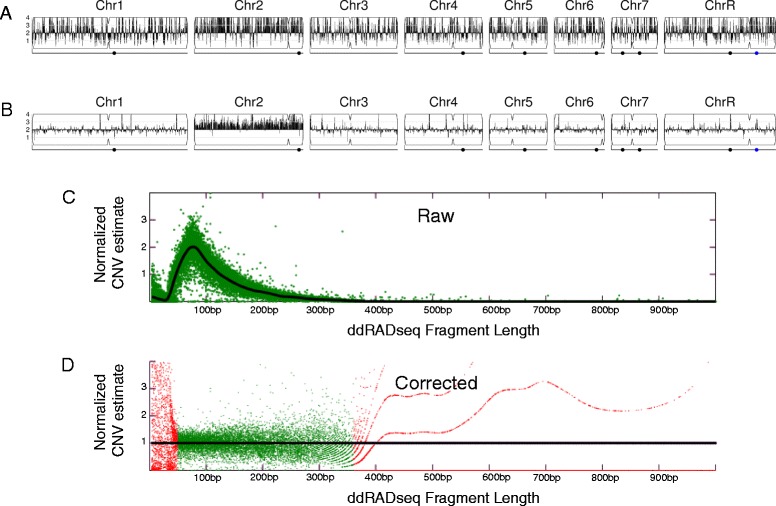
**Normalization of fragment-length-bias in ddRADseq data. (A)** High noise of raw read-depth CNV estimates in CHY477 [35] ddRADseq data with GC-content, fragment-length, and position-effect biases. **(B)** CNV estimates mapped across the genome and corrected for GC bias, fragment length bias and normalized to the reference data. **(C)** Average read-depth CNV estimates versus predicted restriction fragment length for strain RBY917 Mata/a -his, -leu, delta gal1::SAT1/GAL1 derived from SNY87 [36]. Black, LOWESS fit curve. **(D)** Corrected average read-depth CNV estimates versus fragment length, with regions of low reliability data in red, as described in more detail in the text. Chromosome illustrations are as in Figure 2.

CNV analysis of next-generation sequencing data by the pipeline is based upon read depth across the genome. Several biases can impact read depth and thereby interfere with CNV analysis. Two separate biases, a chromosome-end bias and a GC-content bias, appear sporadically in all types of data examined (including microarray and whole genome sequencing (WGseq) data). The mechanism that results in the chromosome end artifact is unclear, but the smooth change in the apparent copy number increase towards the chromosome ends (Figure 2A) suggests that some DNA preparations may release more genomic DNA as a function of telomere proximity (Jane Usher, personal communication). A GC-content bias is due to strong positional variations in GC content in the *C. albicans* genome. This, combined with the PCR amplification bias introduced during sequence library or array preparation, results in a strong positional effect in local copy number estimates (Figure 3A). In datasets produced from the ddRADseq protocol, a third bias is associated with the length of restriction fragments. A fourth bias, seen consistently in all ddRADseq data sets, appears as a high frequency of short-range increases and decreases in read depth at specific genome positions across all strains analyzed, and thus can be removed by normalization to a control dataset from the reference genome. The Y_MAP_ pipeline includes filters, which can be deselected by the user, for each of these biases to correct the data before final presentation and to facilitate detection of *bona fide* CNVs. The final presentation of the corrected copy number data is in the form of a histogram drawn vertically from the figure centerline (Figures 2A,B, 3A,B, and 4A,B).

The chromosome-end bias is normalized using locally weighted scatterplot smoothing (LOWESS) normalization [37] of average read depth versus distance to the nearest chromosome end, for 5,000 bp windows tiled along each chromosome (Figure 2C). The LOWESS fitting is performed with a smoothing window size determined for each dataset as that which produces the least error between the fit and the raw data, using 10-fold cross-validation [38]. Dividing the raw data by the fit curve normalizes the bias (Figure 2D), allowing an unimpeded view of the mapped genome (Figure 2B, a diploid with no significant CNVs). Because this bias is sporadically present, the correction is optional and is not performed by default.

The GC-content bias is normalized using LOWESS normalization of average read depth versus GC content, for 5,000 bp windows tiled along each chromosome (Figure 3C). The LOWESS fitting is performed with a smoothing window size determined for each dataset as that which produces the least error between the fit and the raw data using 10-fold cross-validation. Dividing the raw data by the fit curve normalizes this bias (Figure 3D), allowing an unimpeded visual examination of CNVs across the genome. For example, it can distinguish chromosome number for a near-tetraploid strain with a small segmental duplication near the centromere of ChrR, three copies of chromosomes 4, 5R and 6, and with seven copies of the left arm of chromosome 5R (due to the presence of three copies of whole Chr5 and two copies of an i(5L) with two copies of Chr5L per isochromosome) (Figure 3B). Because this bias is always present to some degree in all data types examined, the correction is performed by default unless deselected by the user.

The ddRADseq protocol generates high read depths at a sub-sampling of genomic loci, resulting in a much-reduced total cost per strain sequenced. The protocol produces a library of restriction fragments digested with two different restriction enzymes (in this case *Mfo*I and *Mpe*I). A strong bias exists in the read depth versus the length of each valid restriction fragment (obtained via a simulated digest of the reference genome, followed by selecting fragments that have the two restriction fragment ends; Figure 4C). The fragment-length-bias is filtered using LOWESS normalization of an average read depth versus the simulated fragment frequency. The LOWESS fitting is performed with a smoothing window size determined for each dataset as that which produces the least error between the fit and the raw data. Restriction fragments less than 50 bp or greater than 1,000 bp show average read depths that exhibit too much noise and are considered unreliable. Where the LOWESS fit line drops below one read, the fragments are considered unreliable due to the reduced dynamic range in the data. These unreliable data are noted (red points in Figure 4D) and not used in later steps of the analysis.

For ddRADseq analyses, first the chromosome-end and GC-content bias corrections are applied using data per valid restriction fragment instead of the standard-sized 5,000 bp windows used in WGseq analysis. After these corrections are performed, there remains a strong position-effect bias in read depth that is uncharacterized. This final bias is corrected by normalizing the corrected read depths for each usable restriction fragment by the corrected read depths from a euploid reference dataset. Because the earlier biases differ from dataset to dataset, the reference normalization is performed as the final normalization step. The result of these corrections is a pronounced reduction in noise in the CNV data as seen by comparing the raw read depth (Figure 4A) to the corrected read depth (Figure 4B) for an example dataset.

After these corrections are applied to the raw sequence read data, the corrected copy number estimates are locally smoothed to reduce the impact of high-frequency noise. The estimates are then multiplied by the whole genome ploidy estimate that was determined by flow cytometry of DNA content and entered during setup of the project. The corrected estimates are plotted as a histogram along each chromosome, with the lines drawn vertically from the baseline ploidy entered during project setup. CNVs are then evident as regions with prominent black bars. A diagram summarizing the flow of information during CNV analysis can be found in Additional file 6.

### SNP/LOH analysis

SNPs are regions of a genome that have two different alleles at the same locus on different homologs. The allelic ratio (0 or 1 for homozygous regions and 0.5 for heterozygous regions in a diploid genome) is used to determine whether a region that had SNPs in the parent/reference strain has undergone LOH to become homozygous. An allelic ratio is calculated for each coordinate by dividing the number of reads with the more abundant base call by the total number of reads at each coordinate (resulting in values ranging from 0.5 to 1.0).

Three styles of analysis are performed, depending on user input during the project setup. The first style is the default option, which is used when no reference strain or hapmap is available. In this case, the SNP distribution for the strain of interest is displayed as vertical grey bars in the background of each chromosome. Once analysis has completed, this strain can be used as the ‘parent’ for other related strains. In the second style of analysis, a parent strain is chosen and the SNPs in common between that parent and the test strain being analyzed are displayed as grey bars (as in the first style), while any SNPs in the parent that have different allelic ratios in the test strain are displayed in red, if allelic ratios approach 0 or 1, or in green, if ratios suggest unusual allele numbers (often due to CNVs or aneuploidy). The third style of analysis can be chosen if a hapmap for the parent strain background is available. SNPs that remain heterozygous are again displayed in grey, while those that have become homozygous are displayed in the color assigned to the homolog that is retained (for example, cyan for the ‘a’ allele and magenta for the ‘b’ allele).

**Figure 5:**
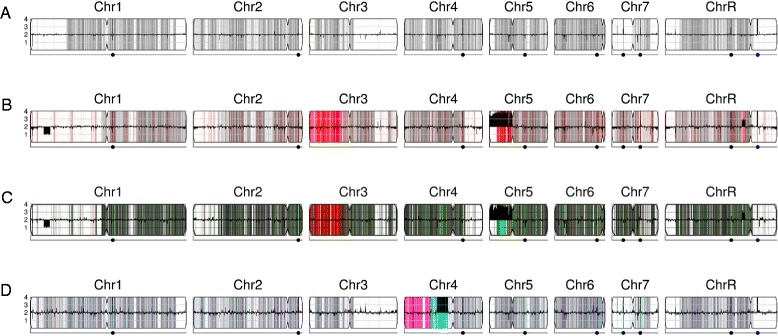
**Presentation styles for WGseq data. (A)** Heterozygous reference strain SC5314 (NCBI Sequence Read Archive (SRA) [39], accession SRR868699) showing SNP density, number of SNPs per 5 kb region illustrated in degree of darkness in grey bars; centromere loci are illustrated as an indentation in the chromosome cartoon. **(B)** Clinical isolate FH5 showing changes in allelic ratio in red and CNV changes including i(5L) in black - all determined relative to the parental strain FH1 (NCBI SRA [40], accession SAMN03144961). **(C)** Strain FH5 relative to strain FH1 (as in **(B)**), with complete LOH in red and allelic ratio changes (for example, 3:1 on Chr5L) in green. **(D)** SC5314-derived lab isolate YJB12746 showing segmental LOH (of both homologs ‘a’ (cyan) and ‘b’ (magenta)) in addition to a segmental aneuploidy on chromosome 4. Chromosome illustrations are as in Figure 2.

For the default option, any coordinates with an allelic ratio near 0.5 (0.50 to 0.75) are considered heterozygous. More extreme allelic ratios are considered to be homozygous, appearing in the dataset due to sequencing errors. The density of heterozygous SNPs is presented as vertical lines spanning the height of each chromosome cartoon, with the intensity of grey color representing the number of SNPs in each 5,000 bp bin. If there are fewer than 100 SNPs in a bin, it is drawn with a lighter shade corresponding to the number of SNPs relative to the 100 SNP threshold. This results in white backgrounds for homozygous regions and increasingly dark shades of grey for regions with higher numbers of SNPs (Figure 5A).

When a parental type strain of unknown genotype (for example, a clinical isolate) is selected for a project, the pipeline first calculates the distribution of SNPs across the parental genome in the manner described above. For comparison of the parental genotype to another related strain (for example, another sample from the same patient), every heterozygous SNP locus in the parent is examined in the second dataset. If the allelic ratio changes from the 0.5 value observed in the reference strain, the SNP is assigned a red color and the final color of each 5,000 bp display bin is calculated as the weighted average of all the SNPs within the bin (Figure 5B). An alternative presentation assigns red color only to coordinates that have transitioned from heterozygous to homozygous (allelic ratio of 1.0) and assigns the green color to coordinates that have unusual allelic ratios (allelic ratios between 0.75 and 1.0, only excluding those with allelic ratios precisely at 1.0) (Figure 5C). Low SNP counts are factored into the presented colors, as described above for the first style of analysis.

When a known hapmap is selected for a project, the pipeline loads SNP coordinates from the map and examines the allelic ratios of the dataset at those coordinates. For disomic regions of the genome, any SNP locus with an allelic ratio near 0.5 (0.50 to 0.75) is considered heterozygous and assigned the color grey. Any SNP locus with a more extreme allelic ratio is considered homozygous and assigned the color corresponding to the homolog with the matching allele in the map. For regions that are monosomic, trisomic, or larger, colors are assigned to SNPs based on the apparent ratio of homologs present. SNPs within each 5,000 bp bin are gathered and the final presented color is determined as the weighted average of the colors assigned to the individual SNPs (Figure 5D). Low SNP counts are factored into the presented colors as in the cases previously described.

**Figure 6:**
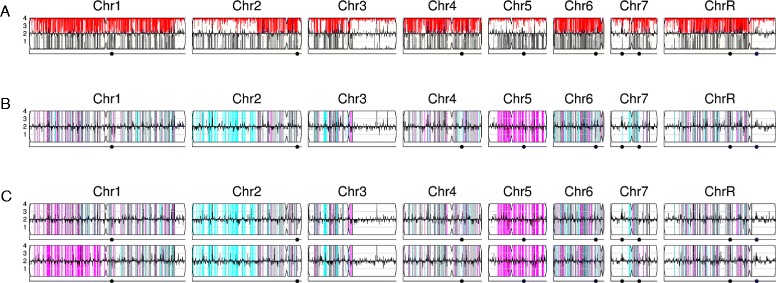
**Presentation styles for ddRADseq data. (A,B)** Allelic ratios drawn as grey lines from top and bottom edges. (A) Allelic ratios for YJB12712 derivative 2 (top, red) compared with reference SC5314 (bottom, grey). Regions that are predominantly white in both samples were homozygous in the parent strain. **(B)** Data from YJB12712 derivative 2 illustrated without the reference control and using the hapmap color scheme: white regions were homozygous in the reference strain, cyan is homolog ‘a’, and magenta is homolog ‘b’. **(C)** Two additional isolates (YJB12712 derivative 1 and YJB12712 derivative 9) from the same experiment illustrating different degrees of LOH on the left arm of Chr1. Chromosome illustrations are as in Figure 2.

The sparse datasets produced from the ddRADseq protocol introduce a high sampling error to allelic ratio calls, increasing the uncertainty of SNP calls and an increased incidence of coordinates that appear as a SNP in one dataset but not another. This sampling error in allelic ratio calls interferes with the direct comparison of SNP loci between a dataset and a parental type dataset. If one dataset is examined without comparison to a reference - producing a very noisy CNV map - the allelic ratios are plotted as grey lines emanating from the top and bottom of each chromosome cartoon inwards to the ratio calculated for each coordinate (where the y-axis ranges from 0.0 to 1.0 for the lines; Figure 6A). When a dataset is examined in comparison with a reference, the pipeline produces a figure with allelic ratios for the reference strain drawn as grey lines emanating from the bottom of the cartoon and allelic ratios for the test dataset plotted as red lines drawn from the top of each chromosome (Figure 6B). Loci with a read-depth lower than 20 are ignored, because the corresponding high sampling error produces a high likelihood of spurious midrange allelic ratios that can appear as heterozygous.

If the user selects a hapmap while setting up an analysis, the higher resolution data of the hapmap allows every SNP locus that appears in the dataset to be examined. The allelic ratios, coupled with the SNP homolog identity information from the hapmap [24,25], allows coordinates to be assigned colors by how consistent they are with either homolog or with the heterozygous state. Lines are then drawn from the top to the bottom of each chromosome for coordinates with allelic ratios less than 1.0, in the color previously assigned (Figure 6C). Allelic ratios of exactly 1.0 are not drawn because they often represent the sampling error found in low read depth areas of the sparse dataset. Visual comparison between the allelic ratio plots for related strains facilitates the identification of large regions of LOH (Figure 6D: magenta at end of left arms of Chr1). A diagram summarizing the flow of information during SNP/LOH analysis can be found in Additional file 7.

### User interface

The Y_MAP_ user interface is implemented in asynchronous Javascript and PHP to ensure a responsive interface that automatically refreshes as aspects of the central computation engine complete. The website allows the user to install new reference genomes and to create ‘projects’ to process raw data. A project in Y_MAP_ is defined as the analysis of a single strain, relative to either a known reference strain (already installed in Y_MAP_) or relative to a user-installed parental/reference genome. In addition, if allelic information is available (from strains that are either haploid or that carry trisomic chromosomes) the website allows construction of hapmaps of such strain backgrounds.

**Figure 7:**
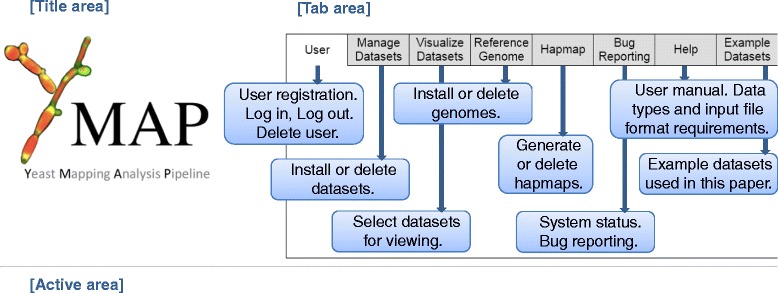
**Outline of user interface to pipeline.** Functions are accessed through the tabbed upper-right portion of the interface. Resulting figures are displayed in the lower portion of the interface.

The main page consists of three distinct areas (Figure 7). The top-left presents the pipeline title and logo. The bottom is an ‘active area’ where dataset result figures are interactively displayed and compared. The top-right area consists of a series of selectable tabbed panels containing the different functions built into Y_MAP_.

The ‘User’ tab contains functions to add and delete users, as well as to log in or out of the system. The ‘Manage Datasets’ tab contains functions to install new projects, as well as functions to display or delete existing projects. Clicking ‘Install New Dataset’, a button located under the main toolbar, loads a page requesting information to define a new project. Inputs required include the name for the new project, the strain ploidy, the baseline ploidy for the generated figures, if annotations are to be drawn in figures, and the data type.

Choosing a data type causes the window to refresh with additional options depending on the data type selected. The data type ‘SNP/CGH microarray’ corresponds to the arrays defined in [25] and only has the option of correcting for the GC bias. This is a new feature, not described in [25], for the analysis of this type of array data. The other data types are all sequence-based and have additional common input requirements; the format of the sequence read data, the choice of the reference genome, the hapmap information (if any) to be used, the parental strain for comparison, and a set of bias-correction filters depending on the type of sequence data. After information about the specific project has been provided on the pop up, the user must click the ‘Create New Dataset’ button at the bottom of the page. This returns the user to the main page. It is then necessary for the user to reload/refresh the main page.

After a dataset has been defined, it is placed in a ‘Datasets Pending’ list at the left side of the tab area. A note is presented below the list indicating the need to wait for any current uploads to complete before reloading the page. To upload the data into the project, the user then clicks on the ‘Add’ button, which appears under the project name as a dark grey colored button. The grey button includes text indicating the expected data type. Selecting the grey upload button will open a file dialog for choosing the file to be uploaded. For paired-end read sequence datasets, a second grey button will appear after the first-end reads file is selected. Once the files are all designated, a green ‘upload’ button appears; clicking this button initiates data upload and analysis. After data files have been uploaded, the color of the dataset name will be changed from red to yellow to indicate the pipeline is processing the data. When the pipeline has completed processing the data, the dataset name will become green. If an unknown file type is uploaded, an error message will be presented. If a dataset is taking longer to process than expected, potentially due to server load or a dataset error, an error message will be presented. Clicking the ‘Delete’ button for a project irreversibly removes it from the site. To avoid inadvertent deletion of uploaded projects, a confirmation is requested from the user.

The ‘Visualize Datasets’ tab allows for the visualization of finished projects in different formats and the window is separated into upper and lower sections. The upper section displays the list of all projects in the user’s account, with the same red/yellow/green color scheme to indicate status. The project data themselves are displayed in the lower section. Once a project is completed, the data can be displayed by checking the checkbox adjacent to the project name, which appears below in the order in which the data display was selected. When an additional project is chosen, an entry for the project is added to the bottom of the display section. The default format is a horizontal figure displaying CNVs and SNPs. Alternative formats (for example, chromosomes displayed horizontally, one above the other) and options to display only CNVs or only SNPs are also available. A displayed project can be removed from the viewing area by clicking the [‘X’] at the top-right of the entry in the lower section of the window. Visualized datasets can be combined into one image by selecting the ‘Combine figures viewed below’ button found below the logo image in the title area at the top-left of the page, then selecting one of the options presented below the button.

The ‘Reference Genome’ tab contains functions to install a reference genome or to delete an installed reference genome. Upon selecting the ‘Install New Genome’ button, a window requests the name of the new genome. The genome name is then placed in the ‘Genomes Pending’ list, with behavior similar to the interface for installing new datasets previously discussed. Selecting the grey upload button opens a file selection dialog, where a FASTA format (or compressed FASTA in ZIP or GZ format) file is to be selected. Importantly, reference genomes should be installed prior to addition of relevant project data, as the uploading/analysis process will ask for the relevant reference genome for the analysis. During installation of a new genome, the loaded FASTA file is first processed to identify the names of included chromosomes. Locations of centromeres, rDNA, any other annotations, as well as any information about open reading frame (ORF) definitions are then loaded and presented in the space below the genome name.

The ‘Hapmap’ tab contains functions for constructing or deleting hapmap definitions. During construction of a new hapmap, the name for the new hapmap, the reference genome, and the first datasets are defined in a window similar to the dataset and genome interfaces. If the hapmap is being constructed from two haploid/homozygous parents, the datasets for those parents are selected in this step. If the hapmap is being constructed from a diploid/heterozygous parent, the parent and a first partially homozygous progeny strain are chosen in this step. For a diploid parent, the next loaded page allows the user to define which regions of the first partially homozygous progeny strain represent an LOH event and which homologs remain. For a diploid or haploid parent, the page also allows the user to choose the colors used to represent the two homologs. The system then processes the datasets and user input to build a hapmap. A hapmap based on a haploid parent will be automatically finalized at this stage; a hapmap based on a diploid parent can be improved with additional datasets by selecting the grey ‘Add haplotype entry…’ button until the user indicates that the hapmap is completed by selecting the grey ‘Finalize haplotype map’ button. More information regarding hapmap generation can be found in Additional file 8.

The ‘Bug Reporting’ tab contains notes about the system status and the option to report bugs to the developers. The ‘Help’ tab contains descriptions of the different input file requirements for the different data types. The ‘Example Datasets’ tab contains files or links to database accessions used to construct the figures in this paper.

## Results and discussion

### Analysis of well-characterized laboratory isolates

**Figure 8:**
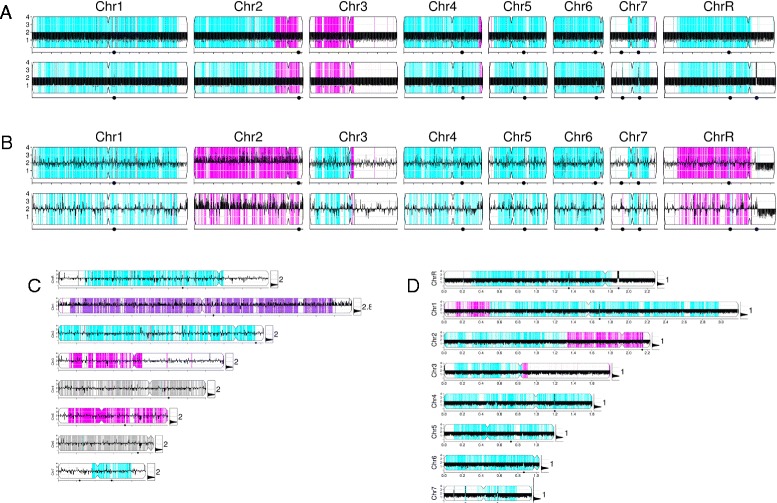
**Analysis of strains derived from** ***C. albicans*** **lab reference strain SC5314. (A)** Comparison of SNP/CGH array (top row) to WGseq (bottom row) for YJB10490, a haploid *C. albicans* derivative of SC5314 [41]. **(B)** Comparison of SNP/CGH-array (top row) to ddRADseq (bottom row) for auto-diploid *C. albicans* strain YJB12229 [41]. **(C)** A SNP/CGH array dataset for near-diploid isolate Ss2 [43], showing LOHs and a trisomy of Chr1. **(D)** WGseq dataset for haploid YJB12353 [41], showing whole-genome LOH.

The Y_MAP_ pipeline has been used to address a number of important questions regarding the dynamics of genome structures. An important feature of Y_MAP_ is the visualization of hapmaps by comparison with a reference WGseq dataset - for example, for comparison of *C. albicans* diploid reference strain SC5314 with a haploid strain derived from it (YJB12353 [41]) using SNP/CGH arrays (Figure 8A). Such haploid genomes were used with the Y_MAP_ hapmap tool to analyze WGseq datasets and to construct a full-resolution hapmap. In this manner, 73,100 SNPs were identified in the SC5314 reference genome. Of these, 222 SNP loci were discarded because of gaps in read coverage, 81 SNP loci were discarded because they did not match either of the reference homologs, and 78 SNP loci were discarded because of the uncertainty in the large LOH region boundaries used to construct the hapmap. In total, 72,729 (99.48% of the reference total) SNP coordinates were mapped to one of the two homologs (Additional file 9), which is comparable to the 69,688 phased SNPs mapped in [42].

The high-resolution hapmap originally constructed with SNP/CGH microarray data [25] and the extended, full-resolution hapmap constructed through the Y_MAP_ pipeline allow direct comparison of datasets from older microarray and WGseq technologies generated when analyzing strains derived from the *C. albicans* reference SC5314. WGseq dataset analysis with the hapmap results in figures (Figure 8A, bottom row) that are nearly indistinguishable from those produced using SNP/CGH microarrays (Figure 8A, top row). The sparse sampling of ddRADseq datasets yields a noisier visualization, but the resulting figures (Figure 8B, bottom row) are also comparable to those produced from array analysis (Figure 8B, top row). In addition to the horizontally arranged genomes illustrated previously, the pipeline outputs figures with chromosomes stacked vertically to maximize the visual discrimination of chromosome-specific changes (Figure 8C,D).

### Analysis of unrelated clinical isolates

**Figure 9:**
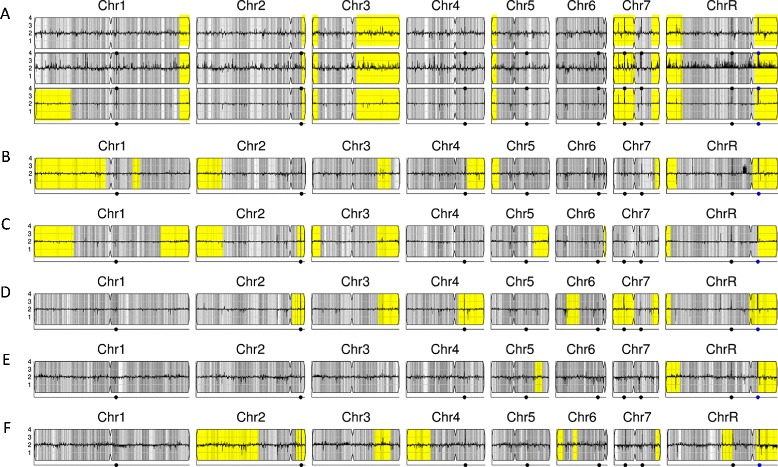
**LOH patterns differ in different** ***C. albicans*** **clinical isolates. (A)** Three isolates of *C. albicans* reference strain C5314 from different sources (EMBL EBI BioSamples [34], accession SAMN02141741; in-house; NCBI SRA, accession SAMN02140351), showing variations. **(B)** FH1. **(C)** ATCC200955 (NCBI SRA [39], accession SAMN02140345). **(D)** ATCC10231 (NCBI SRA [39], accession SAMN02140347). **(E)** YL1 (EMBL EBI BioSamples [34], accession SAMEA1879767). **(F)** YQ2 (EMBL EBI BioSamples [34], accession SAMEA1879786). Grey, heterozygous regions as in previous figures; yellow, regions of contiguous LOH highlighted.

*C. albicans* clinical isolates are highly heterozygous and the majority of the SNPs arose after their divergence from a common ancestor. Individual clinical isolates from different patients also do not have a related parental-type strain to use for comparison. Nonetheless, visualizing SNP density across the genome can reveal evolutionarily recent LOH events. Chromosomal regions with LOH are characterized by very low average SNP density (yellow regions in Figure 9) and differ between unrelated *C. albican*s clinical isolates. For example, reference strain SC5314 (Figure 9A) has large LOHs at the telomeres of chromosomes 3, 7, and R and smaller LOHs at the telomeres of chromosomes 2, 3, and 5 (as illustrated in [40]). Interestingly, other sequencing datasets for SC5314 show additional genome changes, such as aneuploidy and LOH (Figure 9A, middle and lower row). In contrast, clinical isolates from other sources exhibit LOH patterns that differ from SC5314 (Figure 9B-F). Importantly, these simple default style Y_MAP_ cartoons have the power to reveal major differences in the degree of LOH between different isolates. Most, but not all, longer LOH tracts extend to the telomeres, suggestive of single recombination events and/or break-induced replication as the mechanism(s) of homozygosis. Furthermore, while there are some regions that are frequently homozygous (for example, the right arm of ChrR), most of the LOH regions appear to differ between isolates.

### Analysis of serial clinical isolates compared to a parental isolate

In general, most human individuals are thought to be colonized with a single strain of *C. albicans* that they acquired from their mothers [44]. Thus, a related series of clinical isolates collected over the course of treatment in an individual patient can be compared to identify differences acquired over time. Using the Y_MAP_ pipeline, any given isolate can be set as the ‘reference strain’ and data from related isolates can be examined in comparison with this reference WGseq dataset. Essentially, the heterozygous SNPs in the reference are identified and then used as coordinates to be examined for changes in the putative derived isolates. When the hapmap of the reference strain (that is, which SNP alleles are on which homolog) is not known, any SNPs that have become homozygous in the derived isolate are displayed in red, while SNPs that have a large change in allelic ratio are displayed in green. This color scheme allows the rapid discrimination between LOH events and changes in homolog ratios, usually due to aneuploidy.

**Figure 10:**
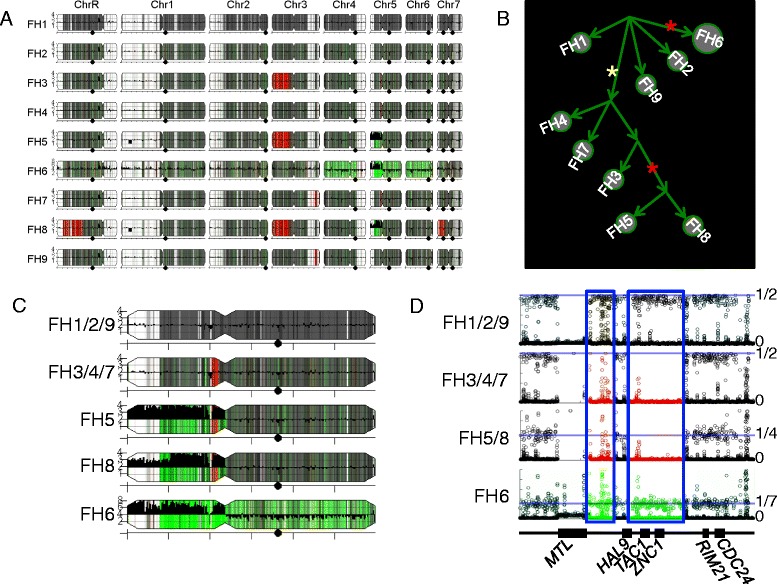
**Comparison of a series of clinical isolates. (A)** Genome maps for the FH series of clinical isolates from an individual patient all compared with the initial isolate (FH1) as in Figure 5C. White, regions homozygous in all isolates; red, regions with recently acquired LOH; green, regions with unusual (neither 1:1 or 1:0) allelic ratios. **(B)** Dendrogram illustrating relationships in FH-series lineage. Yellow star indicates an early *TAC1* LOH event. Red stars indicate independent i(5L) formation events. **(C)** Close-up of Chr5L showing region that underwent LOH event in isolates FH3/4/5/7/8, but not in isolate FH6, using the same color scheme as in **(A)**. **(D)** Allelic ratios surrounding region of Chr5L with LOH (0 = homozygous; 1/2 = heterozygous). Red highlights region of LOH in FH3/4/7/5/8. Horizontal light blue lines indicate expected allelic ratios (top to bottom: 1/2, 1/2, 1/4, and 1/7). Dark blue boxes enclose regions with LOH in FH3/4/5/7/8. Allelic ratio data in the boxes is colored consistent with other subfigures. Mating type locus (MTL) is only found in one copy in assembly 21 of the reference genome. The missing data in the MTL region of FH3/4/5/7/8 indicates these strains are homozygous for the MTL-alpha homolog (not present in the reference genome), while FH1/2/6/9 contain both homologs.

We demonstrate this ability to visualize alterations in SNP distribution using a series of nine isolates collected sequentially over the course of treatment from a patient who developed invasive candidiasis during bone marrow transplant [45]. Isolates (FH1 and FH2) were collected before the patient received fluconazole. During clinical isolation and subsequent culture steps, each isolate experienced at least one single colony bottleneck. Isolate FH1 collected at the earliest time point was used as the parental-type strain. Comparison with the parental type using the pipeline revealed several large and one small LOH tracts across the series (Figure 10), in addition to the copy number changes that were previously characterized using CGH array analysis [2]. A parsimony analysis of the large-scale features (CNV, LOH) that are obviously different between the isolates illustrates the apparent relationships between the series of isolates and how the lineage has evolved over time (Figure 10B; details of the tree in Additional file 10).

The most visually prominent feature in the series is the large LOH of Chr3L, which unites FH3/5/8 into a sub-lineage. FH5/8 share a small segmental deletion on the left arm of chromosome 1 and the presence of an isochromosome (i(5L); red star in Figure 10B), two features not shared by FH3. Interestingly, although isolate FH6 also has an i(5L), it lacks other features of the FH5/8 sub-lineage, including the LOH on Chr5L, indicating that an independent i(5L) formation event occurred in this strain. Consistent with this, FH6 lacks the two small tandem LOH tracts on Chr5L that are found on FH3/4/5/7/8 and that encompass the *TAC1* locus (Figure 10). Furthermore, FH9, a post-mortem tissue sample, is most similar to the initial samples FH1/2, indicating that multiple independent isolates remained in the patient. The complete dendrogram of FH strain relationships (Figure 10B) illustrates the expansion of one sub-lineage after the LOH of *TAC1*. Importantly, the temporal order with which the isolates were collected and numbered does not correlate perfectly with their position on the full lineage. The lack of correlation between collection order and relationship within the inferred lineage is reasonably explained by the sparse sampling of the actual lineage (one colony per time point). A larger number of isolates would be expected to result in a higher correlation, and would capture more of the diversity that developed in the patient during the course of anti-fungal treatment.

## Conclusions

The Y_MAP_ pipeline provides facile conversion of sequence, microarray or ddRADseq data into intuitive genome maps. While the sequence analysis processing steps utilized are generally standard, the assembly of them together in the Y_MAP_ pipeline provides a number of important features collected into one tool: 1) the ability to upload different types of datasets (microarrays, WGseq and ddRADseq); 2) visualization that facilitates the comparison of genome structure between multiple isolates for both copy number and allelic ratio; 3) analysis of well-characterized lab isolates with known haplotypes; 4) analysis of clinical isolates with unknown genome organization; 5) display of CNV and allelic ratio information in one, intuitive vertical plot where the individual chromosomes can be readily distinguished from one another or in horizontal plots to facilitate isolate comparisons; and 6) web accessibility that does not require a particular local operating system. In addition, unlike many available databases, Y_MAP_ is designed to accept genomic data for different species and it can build hapmaps for those genomes if the data for assigning alleles are available.

Future developments are planned to permit the import of IonTorrent sequencing data, RNAseq data sets, and ChIPseq data to map positions of DNA binding proteins. We also envision modification of the pipeline to enable output of SNP and CNV data to a GBrowse format that operates on the Stanford genome database and Candida Genome Database [46] for the facile comparison of datasets with the comprehensive gene annotations available for the *C. albicans* and other *Candida* species at the Candida Genome Database. Finally, we are continuing to add the ability to input data from different genomes, including those of *Candida glabrata*, *Candida tropicalis*, and *Candida dubliniensis.*

## Availability and requirements

- Project name: Yeast Mapping Analysis Pipeline (Y_MAP_ )
- Project home page: [28]
- Operating systems: Platform independent.
- Programming languages: Javascript (v1.5+), PHP (v5.3.10), Python (v2.7.3), Matlab R2012a (v7.14.0.739), GNU-bash shell (v4.2.25).

Other requirements:

- Client-side software: Blink- (Google Chrome, Opera, etc.) or WebKit- (Safari, etc.) based web browser.
- Server-side software: GNU-bash (v4.2.25), Java6, Java7, Bowtie2 (v2.1.0), Samtools (v0.1.18), FASTQC (v0.10.1), GATK (v2.8-1), PicardTools (v1.105), and Seqtk.
- License: MIT license [47]
- Any restrictions to use by non-academics: one of the programs used by the pipeline (GATK) requires a license for commercial use.

## Additional files

Additional file 1: Figure S1. Will Y_MAP_ be of use to you? **(A)** Flow diagram to help determine if Y_MAP_ pipeline will be able to analyze your data. **(B)** Flow diagram to help determine if Y_MAP_ pipeline will be able to construct a hapmap from your data. Additional file 2: Figure S2. New project installation. Flow diagram and input needed by Y_MAP_ pipeline to install a new project for analysis. Additional file 3: Figure S3. New reference genome installation. Flow diagram and input needed by Y_MAP_ pipeline to install a new reference genome. Additional file 4: Figure S4. New hapmap construction. Flow diagram and input needed by Y_MAP_ pipeline to construct a new hapmap from analyzed project datasets. Additional file 5: Figure S5. Developmental view of new genome installation. Diagram following information flow during installation and processing of a new reference genome in the Y_MAP_ pipeline backend. Additional file 6: Figure S6. Developmental view of CNV analysis. Diagram following information flow during CNV analysis of a new project dataset in the Y_MAP_ pipeline backend. Additional file 7: Figure S7. Developmental view of SNP/LOH analysis. Diagram following information flow during SNP/LOH analysis of a new project dataset in the Y_MAP_ pipeline backend. Additional file 8: Figure S8. Developmental view of hapmap generation. Diagram following information flow during generation of a new hapmap in the Y_MAP_ pipeline backend. **(A)** Making a hapmap from two haploid/homozygous references. **(B)** Making a hapmap from one heterozygous diploid reference. Additional file 9: Table S1. Full-resolution hapmap. A tab-delimited text file of the hapmap constructed for SC5314 and derived strains using the Y_MAP_ pipeline. Additional file 10: Figure S9. Detailed dendrogram of FH series lineage. Descriptions of features used in parsimony analysis during lineage construction. **(a)** Small LOH Chr1 (at approximately 3 Mb) and Chr7 (at approximately 0.4 Mb). **(b)** 2n - > 4n, +i5L*2, ∆Chr4, ∆Chr5, ∆Chr6. **(c)** Small LOH Chr1 (at approximately 1.5 Mb), small LOH Chr3 (at approximately 0.75 Mb). **(d)** Large LOH Chr3 (at approximately 1.6 Mb to 1.8 Mb). **(e)** Tandem small LOH Chr5 (at approximately 0.4 Mb). **(f)** Small LOH Chr3 (at approximately 1.75 Mb). **(g)** Large LOH Chr3 (at approximately 1.4 Mb to 1.8 Mb). **(h)** Small LOH Chr2 (at approximately 1 Mb), large LOH Chr3 (approximately 0 to 0.7 Mb). **(i)** Segmental ∆Chr1 (approximately 0.3 to 0.4 Mb), +i5L. **(j)** Small LOH Chr3 (at approximately 0.5 Mb). **(k)** Large LOH Chr7 (0 to approximately 0.3 Mb), large LOH ChrR (approximately 0 to 1 Mb), segmental ∆Chr5L (0.0 to approximately 0.2 Mb).

## Abbreviations

BAM: Binary sequence Alignment/Mapping
bp: base pair
CGH: comparative genomic hybridization
CNV: copy number variation
ddRADseq: double digest restriction site associated DNA sequencing
GATK: Genome Analysis ToolKit
LOH: loss of heterozygosity
SAM: Sequence Alignment/Mapping
SNP: single nucleotide polymorphism
SRA: Sequence Read Archive
WGseq: whole genome sequencing
