# Supplementary figures and images for: YMAP: a pipeline for visualization of copy number variation and loss of heterozygosity in eukaryotic pathogens
Source: Genome Med. 2014 Nov 20;6(11):100. doi: 10.1186/s13073-014-0100-8 (PMC4263066; doi:10.1186/s13073-014-0100-8)

## Slide 1
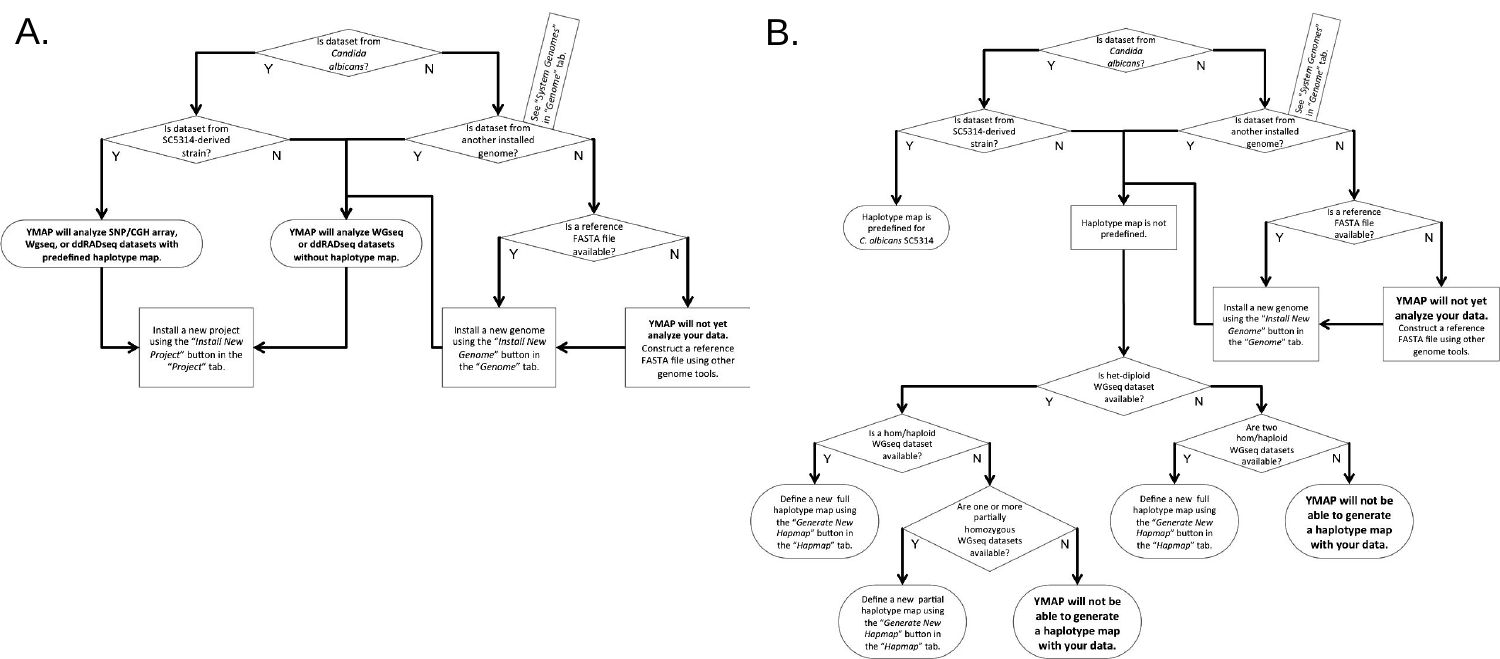

A.
B.

Supplement: Additional file 1: Figure S1. — Will YMAP be of use to you? (A) Flow diagram to help determine if YMAP pipeline will be able to analyze your data. (B) Flow diagram to help determine if YMAP pipeline will be able to construct a hapmap from your data. [file 13073_2014_100_MOESM1_ESM.pptx]

## Slide 1
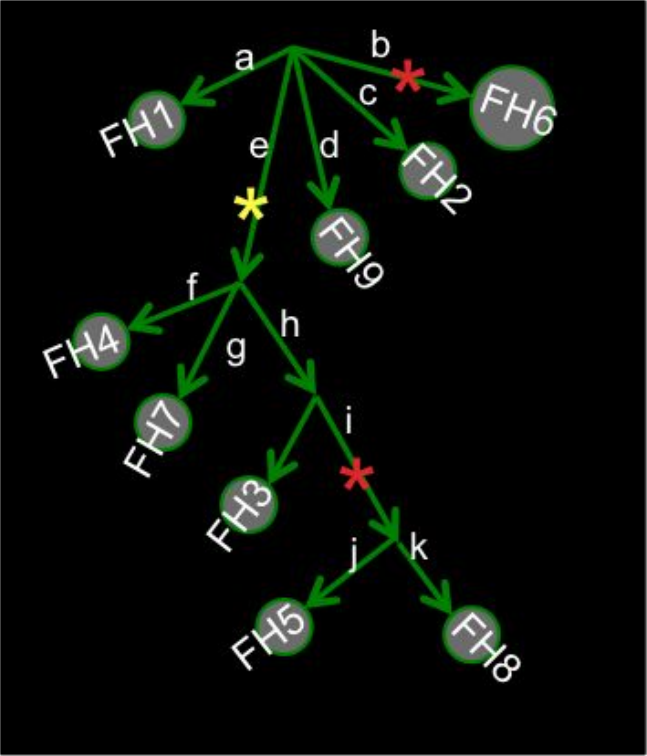

Supplement: Additional file 10: Figure S9. — Detailed dendrogram of FH series lineage. Descriptions of features used in parsimony analysis during lineage construction. (a) Small LOH Chr1 (at approximately 3 Mb) and Chr7 (at approximately 0.4 Mb). (b) 2n - > 4n, +i5L*2, ∆Chr4, ∆Chr5, ∆Chr6. (c) Small LOH Chr1 (at approximately 1.5 Mb), small LOH Chr3 (at approximately 0.75 Mb). (d) Large LOH Chr3 (at approximately 1.6 Mb to 1.8 Mb). (e) Tandem small LOH Chr5 (at approximately 0.4 Mb). (f) Small LOH Chr3 (at approximately 1.75 Mb). (g) Large LOH Chr3 (at approximately 1.4 Mb to 1.8 Mb). (h) Small LOH Chr2 (at approximately 1 Mb), large LOH Chr3 (approximately 0 to 0.7 Mb). (i) Segmental ∆Chr1 (approximately 0.3 to 0.4 Mb), +i5L. (j) Small LOH Chr3 (at approximately 0.5 Mb). (k) Large LOH Chr7 (0 to approximately 0.3 Mb), large LOH ChrR (approximately 0 to 1 Mb), segmental ∆Chr5L (0.0 to approximately 0.2 Mb). [file 13073_2014_100_MOESM10_ESM.pptx]
