# Supplementary material for: YMAP: a pipeline for visualization of copy number variation and loss of heterozygosity in eukaryotic pathogens
Source: Genome Med. 2014 Nov 20;6(11):100. doi: 10.1186/s13073-014-0100-8 (PMC4263066; doi:10.1186/s13073-014-0100-8)
Supplement: Additional file 2: Figure S2. — New project installation. Flow diagram and input needed by YMAP pipeline to install a new project for analysis. [file 13073_2014_100_MOESM2_ESM.pptx]

## Slide 1
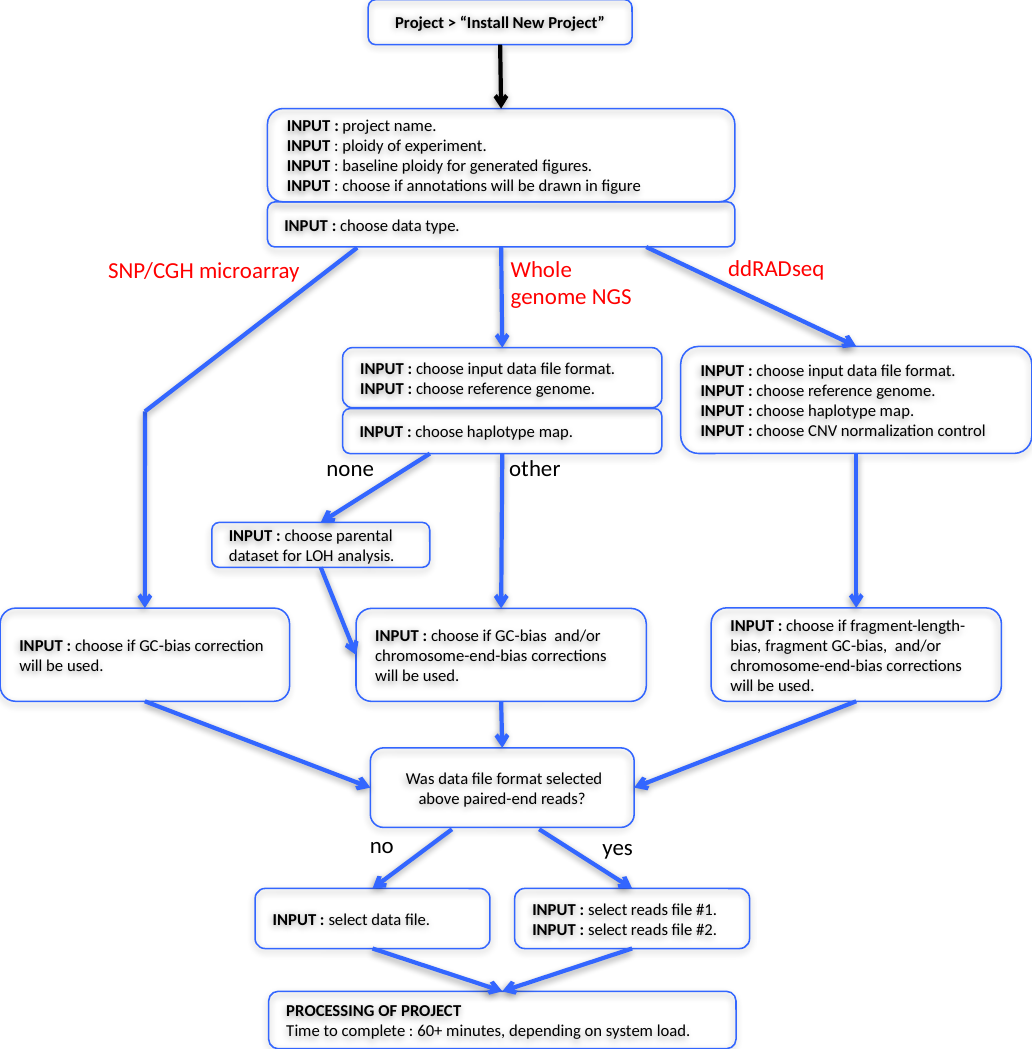

Project > “Install New Project”
INPUT : project name.
INPUT : ploidy of experiment.
INPUT : baseline ploidy for generated figures.
INPUT : choose if annotations will be drawn in figure
INPUT : choose data type.
ddRADseq
Whole genome NGS
SNP/CGH microarray
INPUT : choose input data file format.
INPUT : choose reference genome.
INPUT : choose haplotype map.
INPUT : choose CNV normalization control
INPUT : choose input data file format.
INPUT : choose reference genome.
INPUT : choose haplotype map.
none
other
INPUT : choose parental dataset for LOH analysis.
INPUT : choose if fragment-length-bias, fragment GC-bias, and/or chromosome-end-bias corrections will be used.
INPUT : choose if GC-bias correction will be used.
INPUT : choose if GC-bias and/or chromosome-end-bias corrections will be used.
 Was data file format selected above paired-end reads?
no
yes
INPUT : select data file.
INPUT : select reads file #1.
INPUT : select reads file #2.
PROCESSING OF PROJECT
Time to complete : 60+ minutes, depending on system load.
