# Supplementary material for: YMAP: a pipeline for visualization of copy number variation and loss of heterozygosity in eukaryotic pathogens
Source: Genome Med. 2014 Nov 20;6(11):100. doi: 10.1186/s13073-014-0100-8 (PMC4263066; doi:10.1186/s13073-014-0100-8)
Supplement: Additional file 3: Figure S3. — New reference genome installation. Flow diagram and input needed by YMAP pipeline to install a new reference genome. [file 13073_2014_100_MOESM3_ESM.pptx]

## Slide 1
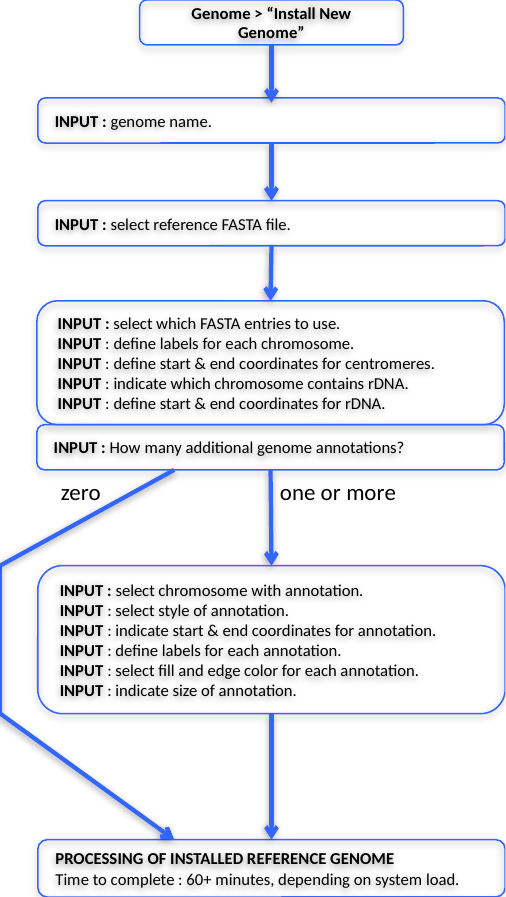

Genome > “Install New Genome”
INPUT : genome name.
INPUT : select reference FASTA file.
INPUT : select which FASTA entries to use.
INPUT : define labels for each chromosome.
INPUT : define start & end coordinates for centromeres.
INPUT : indicate which chromosome contains rDNA.
INPUT : define start & end coordinates for rDNA.
INPUT : How many additional genome annotations?
zero
one or more
INPUT : select chromosome with annotation.
INPUT : select style of annotation.
INPUT : indicate start & end coordinates for annotation.
INPUT : define labels for each annotation.
INPUT : select fill and edge color for each annotation.
INPUT : indicate size of annotation.
PROCESSING OF INSTALLED REFERENCE GENOME
Time to complete : 60+ minutes, depending on system load.
