# Supplementary material for: YMAP: a pipeline for visualization of copy number variation and loss of heterozygosity in eukaryotic pathogens
Source: Genome Med. 2014 Nov 20;6(11):100. doi: 10.1186/s13073-014-0100-8 (PMC4263066; doi:10.1186/s13073-014-0100-8)
Supplement: Additional file 4: Figure S4. — New hapmap construction. Flow diagram and input needed by YMAP pipeline to construct a new hapmap from analyzed project datasets. [file 13073_2014_100_MOESM4_ESM.pptx]

## Slide 1
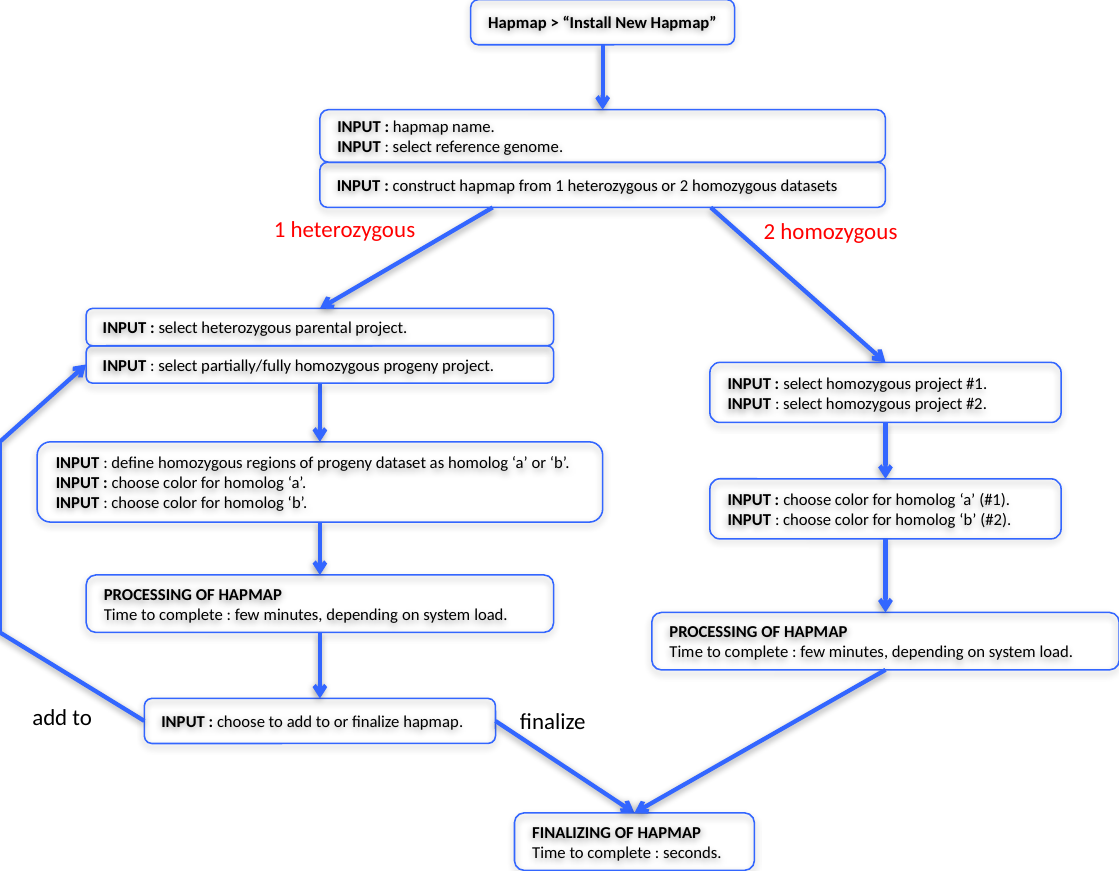

Hapmap > “Install New Hapmap”
INPUT : hapmap name.
INPUT : select reference genome.
INPUT : construct hapmap from 1 heterozygous or 2 homozygous datasets
1 heterozygous
2 homozygous
INPUT : select heterozygous parental project.
INPUT : select partially/fully homozygous progeny project.
INPUT : select homozygous project #1.
INPUT : select homozygous project #2.
INPUT : define homozygous regions of progeny dataset as homolog ‘a’ or ‘b’.
INPUT : choose color for homolog ‘a’.
INPUT : choose color for homolog ‘b’.
INPUT : choose color for homolog ‘a’ (#1).
INPUT : choose color for homolog ‘b’ (#2).
PROCESSING OF HAPMAP
Time to complete : few minutes, depending on system load.
PROCESSING OF HAPMAP
Time to complete : few minutes, depending on system load.
add to
INPUT : choose to add to or finalize hapmap.
finalize
FINALIZING OF HAPMAP
Time to complete : seconds.
