# Supplementary material for: YMAP: a pipeline for visualization of copy number variation and loss of heterozygosity in eukaryotic pathogens
Source: Genome Med. 2014 Nov 20;6(11):100. doi: 10.1186/s13073-014-0100-8 (PMC4263066; doi:10.1186/s13073-014-0100-8)
Supplement: Additional file 5: Figure S5. — Developmental view of new genome installation. Diagram following information flow during installation and processing of a new reference genome in the YMAP pipeline backend. [file 13073_2014_100_MOESM5_ESM.pptx]

## Slide 1
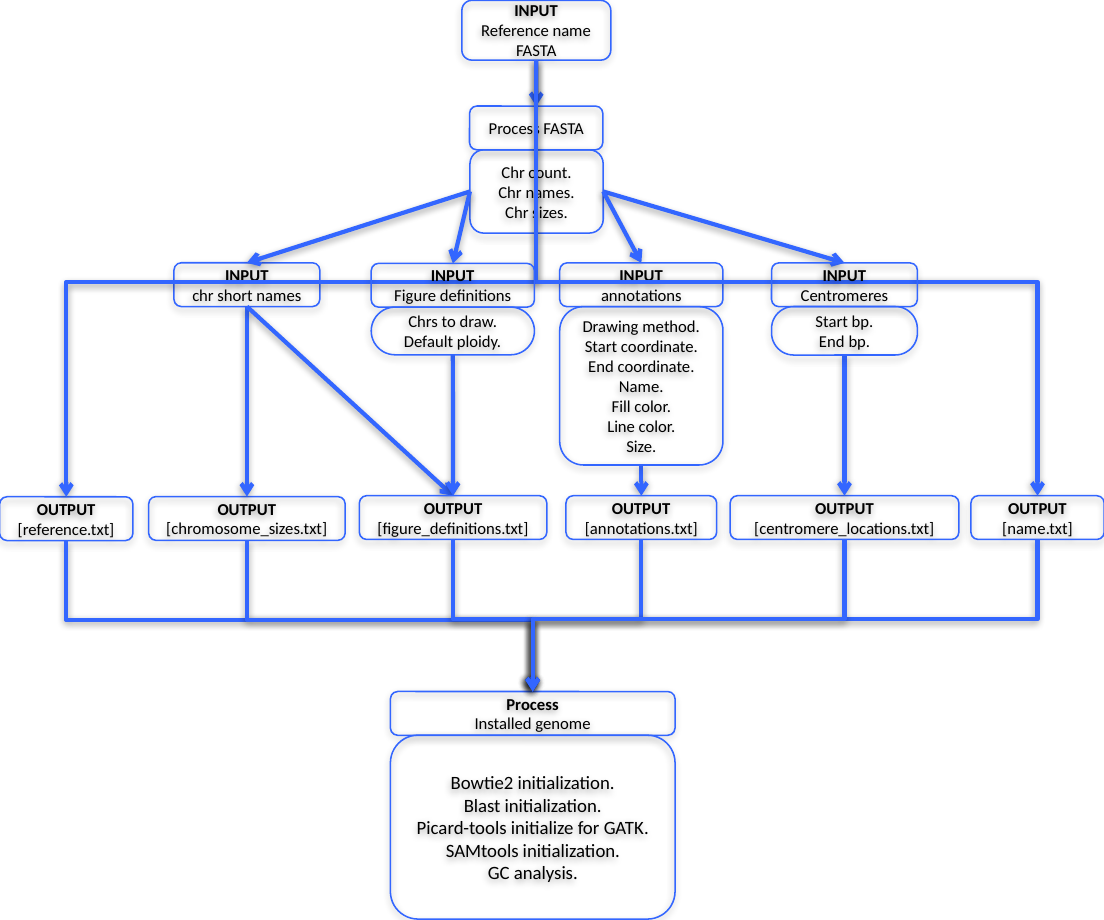

INPUT
Reference name
FASTA
Process FASTA
Chr count.
Chr names.
Chr sizes.
INPUT
chr short names
INPUT
annotations
Drawing method.
Start coordinate.
End coordinate.
Name.
Fill color.
Line color.
Size.
INPUT
Centromeres
Start bp.
End bp.
INPUT
Figure definitions
Chrs to draw.
Default ploidy.
OUTPUT
[figure_definitions.txt]
OUTPUT
[annotations.txt]
OUTPUT
[centromere_locations.txt]
OUTPUT
[name.txt]
OUTPUT
[chromosome_sizes.txt]
OUTPUT
[reference.txt]
Process
Installed genome
Bowtie2 initialization.
Blast initialization.
Picard-tools initialize for GATK.
SAMtools initialization.
GC analysis.
