# Supplementary material for: YMAP: a pipeline for visualization of copy number variation and loss of heterozygosity in eukaryotic pathogens
Source: Genome Med. 2014 Nov 20;6(11):100. doi: 10.1186/s13073-014-0100-8 (PMC4263066; doi:10.1186/s13073-014-0100-8)
Supplement: Additional file 6: Figure S6. — Developmental view of CNV analysis. Diagram following information flow during CNV analysis of a new project dataset in the YMAP pipeline backend. [file 13073_2014_100_MOESM6_ESM.pptx]

## Slide 1
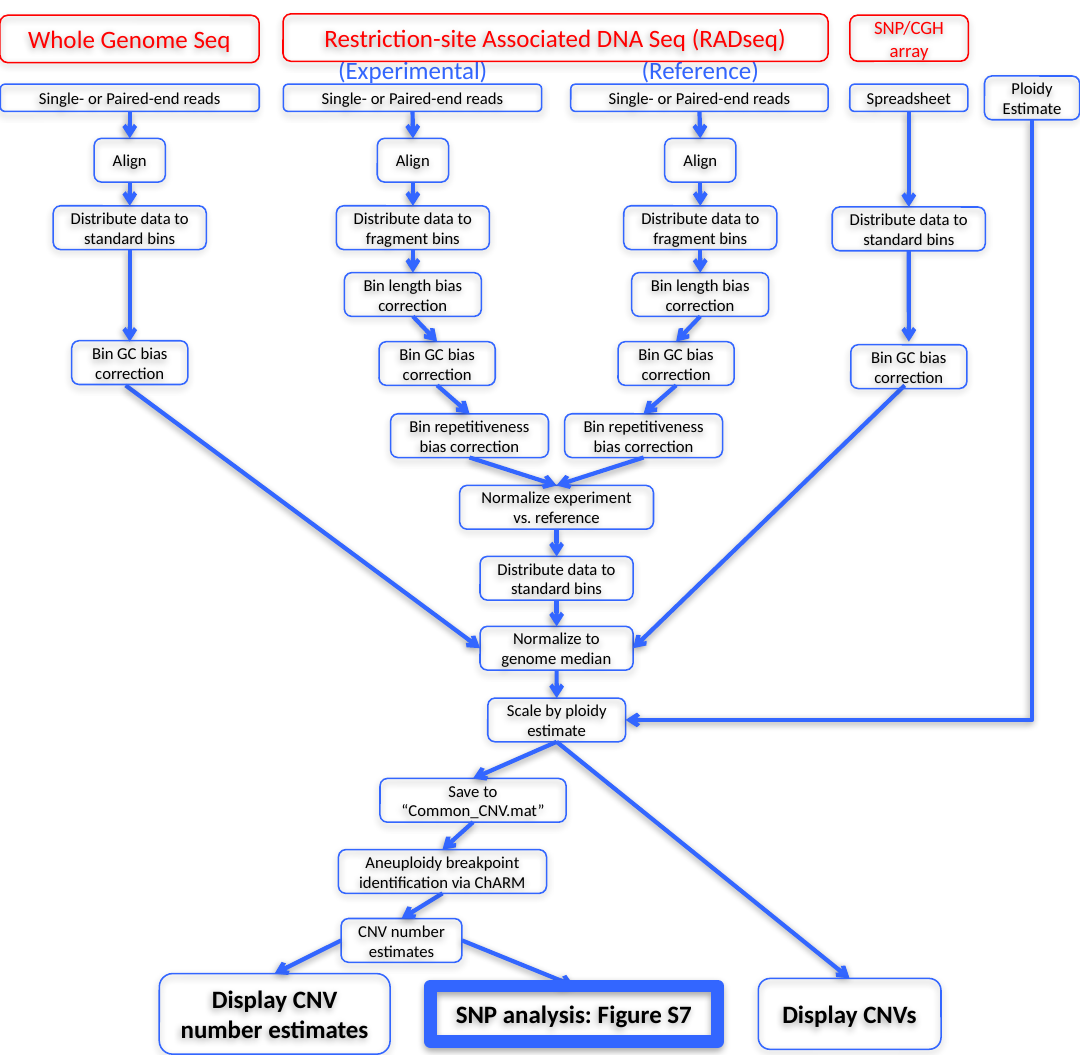

Restriction-site Associated DNA Seq (RADseq)
SNP/CGH
array
Whole Genome Seq
(Experimental)
(Reference)
Ploidy Estimate
Single- or Paired-end reads
Single- or Paired-end reads
Single- or Paired-end reads
Spreadsheet
Align
Align
Align
Distribute data to standard bins
Distribute data to fragment bins
Distribute data to fragment bins
Distribute data to standard bins
Bin length bias correction
Bin length bias correction
Bin GC bias correction
Bin GC bias correction
Bin GC bias correction
Bin GC bias correction
Bin repetitiveness bias correction
Bin repetitiveness bias correction
Normalize experiment vs. reference
Distribute data to standard bins
Normalize to genome median
Scale by ploidy estimate
Save to “Common_CNV.mat”
Aneuploidy breakpoint identification via ChARM
CNV number estimates
Display CNV number estimates
Display CNVs
SNP analysis: Figure S7
