# Supplementary material for: YMAP: a pipeline for visualization of copy number variation and loss of heterozygosity in eukaryotic pathogens
Source: Genome Med. 2014 Nov 20;6(11):100. doi: 10.1186/s13073-014-0100-8 (PMC4263066; doi:10.1186/s13073-014-0100-8)
Supplement: Additional file 7: Figure S7. — Developmental view of SNP/LOH analysis. Diagram following information flow during SNP/LOH analysis of a new project dataset in the YMAP pipeline backend. [file 13073_2014_100_MOESM7_ESM.pptx]

## Slide 1
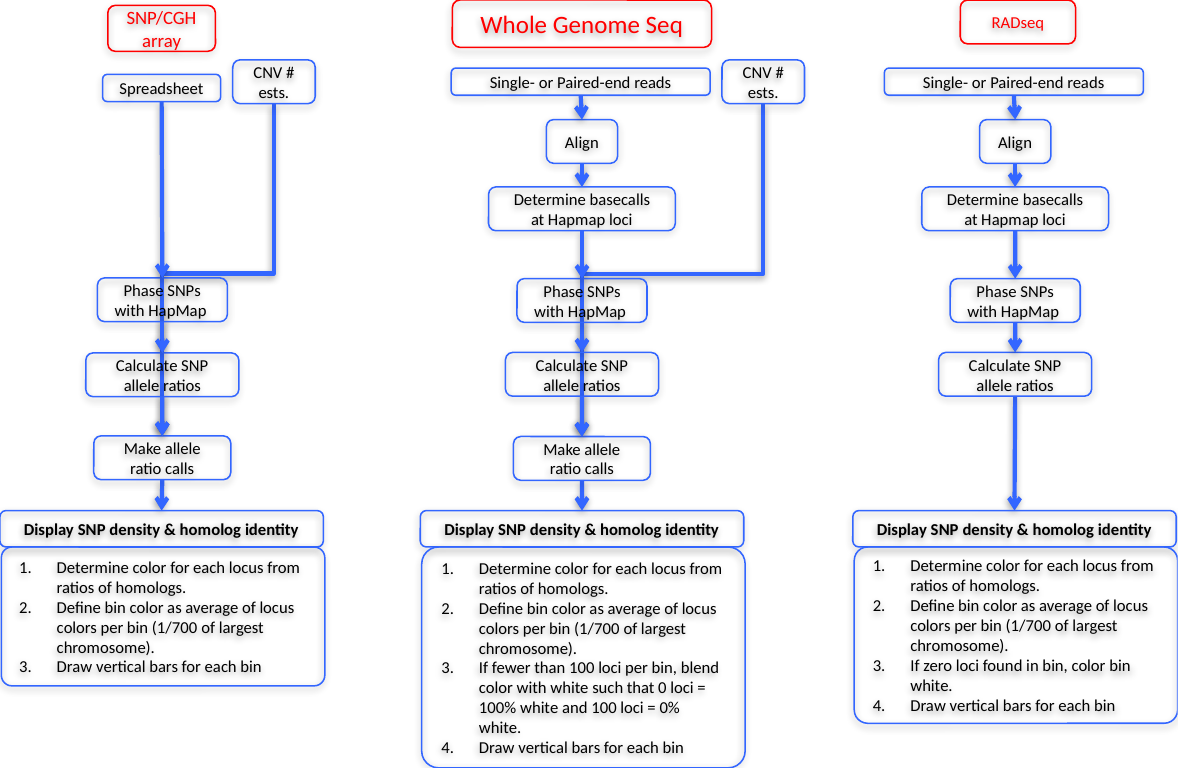

Whole Genome Seq
RADseq
SNP/CGH
array
CNV # ests.
CNV # ests.
Single- or Paired-end reads
Single- or Paired-end reads
Spreadsheet
Align
Align
Determine basecalls at Hapmap loci
Determine basecalls at Hapmap loci
Phase SNPs with HapMap
Phase SNPs with HapMap
Phase SNPs with HapMap
Calculate SNP allele ratios
Calculate SNP allele ratios
Calculate SNP allele ratios
Make allele ratio calls
Make allele ratio calls
Display SNP density & homolog identity
Display SNP density & homolog identity
Display SNP density & homolog identity
Determine color for each locus from ratios of homologs.
Define bin color as average of locus colors per bin (1/700 of largest chromosome).
If fewer than 100 loci per bin, blend color with white such that 0 loci = 100% white and 100 loci = 0% white.
Draw vertical bars for each bin
Determine color for each locus from ratios of homologs.
Define bin color as average of locus colors per bin (1/700 of largest chromosome).
Draw vertical bars for each bin
Determine color for each locus from ratios of homologs.
Define bin color as average of locus colors per bin (1/700 of largest chromosome).
If zero loci found in bin, color bin white.
Draw vertical bars for each bin
