# Supplementary material for: YMAP: a pipeline for visualization of copy number variation and loss of heterozygosity in eukaryotic pathogens
Source: Genome Med. 2014 Nov 20;6(11):100. doi: 10.1186/s13073-014-0100-8 (PMC4263066; doi:10.1186/s13073-014-0100-8)
Supplement: Additional file 8: Figure S8. — Developmental view of hapmap generation. Diagram following information flow during generation of a new hapmap in the YMAP pipeline backend. (A) Making a hapmap from two haploid/homozygous references. (B) Making a hapmap from one heterozygous diploid reference. [file 13073_2014_100_MOESM8_ESM.pptx]

## Slide 1
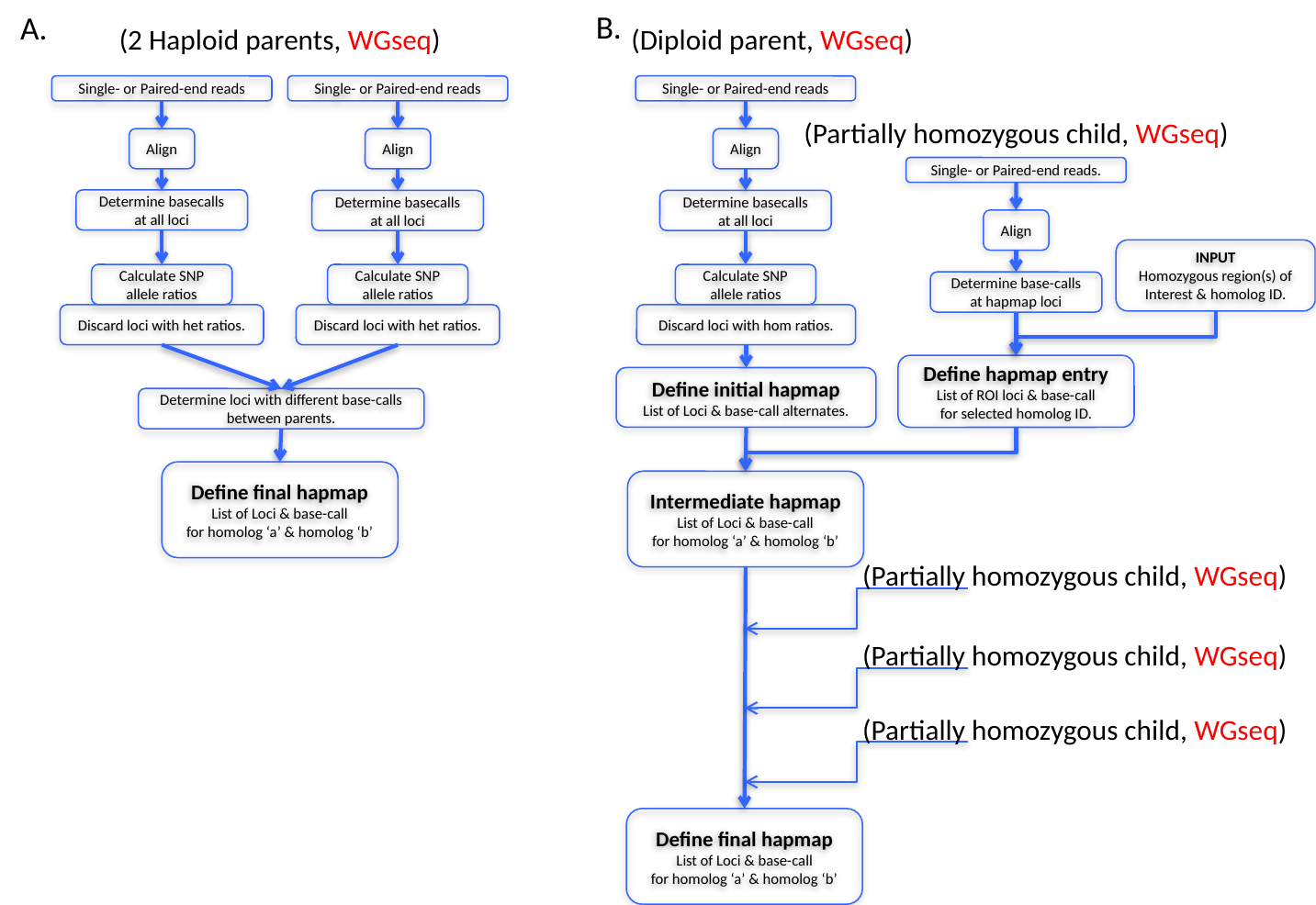

B.
A.
(Diploid parent, WGseq)
(2 Haploid parents, WGseq)
Single- or Paired-end reads
Single- or Paired-end reads
Single- or Paired-end reads
(Partially homozygous child, WGseq)
Align
Align
Align
Single- or Paired-end reads.
Determine basecalls at all loci
Determine basecalls at all loci
Determine basecalls at all loci
Align
INPUT
Homozygous region(s) of
Interest & homolog ID.
Calculate SNP allele ratios
Calculate SNP allele ratios
Calculate SNP allele ratios
Determine base-calls at hapmap loci
Discard loci with het ratios.
Discard loci with het ratios.
Discard loci with hom ratios.
Define hapmap entry
List of ROI loci & base-call
for selected homolog ID.
Define initial hapmap
List of Loci & base-call alternates.
Determine loci with different base-calls between parents.
Define final hapmap
List of Loci & base-call
for homolog ‘a’ & homolog ‘b’
Intermediate hapmap
List of Loci & base-call
for homolog ‘a’ & homolog ‘b’
(Partially homozygous child, WGseq)
(Partially homozygous child, WGseq)
(Partially homozygous child, WGseq)
Define final hapmap
List of Loci & base-call
for homolog ‘a’ & homolog ‘b’
